# Profound Effects of Dexamethasone on the Immunological State, Synthesis and Secretion Capacity of Human Testicular Peritubular Cells

**DOI:** 10.3390/cells11193164

**Published:** 2022-10-09

**Authors:** Youli Konstantinovitch Stepanov, Jan Dominik Speidel, Carola Herrmann, Nina Schmid, Rüdiger Behr, Frank-Michael Köhn, Jan Bernd Stöckl, Ulrich Pickl, Matthias Trottmann, Thomas Fröhlich, Artur Mayerhofer, Harald Welter

**Affiliations:** 1 Laboratory for Functional Genome Analysis LAFUGA, Gene Center, LMU München, 81377 München, Germany; 2 Biomedical Center, Cell Biology, Anatomy III, Faculty of Medicine, Ludwig Maximilian University Munich, 82152 Planegg-Martinsried, Germany; 3 Platform Degenerative Diseases, German Primate Center, Leibniz Institute for Primate Research, 37077 Göttingen, Germany; 4 Andrologicum, 80331 Munich, Germany; 5 Urologie und Andrologie, 80333 Munich, Germany

**Keywords:** human male fertility, human testis, dexamethasone, cytokines, proteomics

## Abstract

The functions of human testicular peritubular cells (HTPCs), forming a small compartment located between the seminiferous epithelium and the interstitial areas of the testis, are not fully known but go beyond intratesticular sperm transport and include immunological roles. The expression of the glucocorticoid receptor (GR) indicates that they may be regulated by glucocorticoids (GCs). Herein, we studied the consequences of the GC dexamethasone (Dex) in cultured HTPCs, which serves as a unique window into the human testis. We examined changes in cytokines, mainly by qPCR and ELISA. A holistic mass-spectrometry-based proteome analysis of cellular and secreted proteins was also performed. Dex, used in a therapeutic concentration, decreased the transcript level of proinflammatory cytokines, e.g., *IL6*, *IL8* and *MCP1*. An siRNA-mediated knockdown of GR reduced the actions on *IL6.* Changes in IL6 were confirmed by ELISA measurements. Of note, Dex also lowered GR levels. The proteomic results revealed strong responses after 24 h (31 significantly altered cellular proteins) and more pronounced ones after 72 h of Dex exposure (30 less abundant and 42 more abundant cellular proteins). Dex also altered the composition of the secretome (33 proteins decreased, 13 increased) after 72 h. Among the regulated proteins were extracellular matrix (ECM) and basement membrane components (e.g., FBLN2, COL1A2 and COL3A1), as well as PTX3 and StAR. These results pinpoint novel, profound effects of Dex in HTPCs. If transferrable to the human testis, changes specifically in ECM and the immunological state of the testis may occur in men upon treatment with Dex for medical reasons.

## 1. Introduction

The glucocorticoid receptor (GR; *NR3C1*, nuclear receptor subfamily 3 group C member 1) is present in the human testis [1], but its involvement in the regulation of testicular functions is not well established. This topic remains difficult to study in men, yet elevated levels of glucocorticoids (GCs) impact the human testis [2,3], as can also be deduced from impaired testicular functions in patients with Cushing′s disease [2,4,5]. Although pertinent information is very limited, lower GC levels may also impair testicular functions in men [6]. To what degree direct testicular effects of GC and/or indirect ones, i.e., via hypothalamic–pituitary inhibition, are responsible is not known.

Recent publications revealed the cellular localization of NR3C1 in the human testis [7,8] and indicated its presence specifically in all peritubular (myoid) cells, i.e., the cells, which, together with the extracellular matrix (ECM) form a small compartment located between the seminiferous epithelium and the interstitial compartment. Human testicular peritubular (HTPCs) cells can be isolated [9,10,11] and because the in situ characteristics of peritubular cells are well preserved in vitro [10,12,13], studies in HTPCs open an experimental window to the human testis.

A recent study showed that GCs target HTPCs and can alter the cellular phenotype [8]. The addition of dexamethasone (Dex), a synthetic GR agonist, caused the translocation of the GR into the nucleus. The consequences of GR activation were increased levels of the typical genes expressed by HTPCs, namely smooth muscle markers (smooth muscle actin (ACTA2)). Cytoskeletal ACTA2-rearrangements were observed and were associated with an increased ability to contract, indicating that GCs are likely involved in the maintenance of the contractile, smooth muscle-like phenotype of peritubular cells. This feature of HTPCs, which enables contractions and relaxation cycles, is crucial for the intratesticular transport of sperm [11,14,15].

The testis possesses a unique immune environment, and previous studies in HTPCs revealed that these cells contribute to this environment e.g., by producing cytokines [16,17,18]. Thus, they are an element of the immune surveillance of the testis [19], which maintains testicular homeostasis and presumably influences male infertility. GCs are well known for their immune-suppressive roles [20,21,22,23], and we therefore hypothesized that they may be involved in the immunological activities of HTPCs as well. Recent studies further report that immune regulation by GCs is linked to tissue and cell type-dependent responses [24,25,26,27]. HTPCs have been recognized to have further important functions and are involved in the regulation of spermatogenesis and testicular paracrine communication [10,28].

We designed this study, in which we combined a targeted approach, focusing mainly on cytokines, and an untargeted proteomic approach to determine the consequences of Dex on the cellular proteome and the secretome of HTPCs.

## 2. Materials and Methods

### 2.1. Human Samples, Cell Culture and Reagents

Human testicular peritubular cells (HTPCs) were isolated as described earlier [9]. They were obtained from small testicular biopsies of men (29–55 years), mainly from patients undergoing TESE (testicular sperm extraction). The identity and purity of cells were assessed, as described [9]. The local Ethics Committee (Technical University of Munich, Faculty of Medicine; project 491/18S-KK) approved the study and the scientific use of the cells. Written informed consent from all patients was obtained. The experiments were carried out in accordance with the relevant guidelines and regulations. For all experiments, freshly isolated or cryopreserved HTPCs from passages 7–12 were used. Cells were cultured in Dulbecco´s modified Eagle Medium (containing high glucose (4.5 g/L), DMEM; Gibco, Paisley, UK) with 10% (*v/v*) fetal calf serum (FCS; Capricorn Scientific, Ebsdorfergrund, Germany) and 1% (*w/v*) penicillin/streptomycin (P/S; Gibco, Paisley, UK) at 37 °C, 5 % CO_2_ and 95 % humidity [1,29].

### 2.2. Animal Samples, Isolation and Cultivation of Mktpcs

The isolation and culture of monkey testicular peritubular cells (MKTPCs) from Common marmoset monkeys (*Callithrix jacchus*) were previously described [30]. We used cryopreserved MKTPCs (passages 2–3), for the experiments. In brief, cells stemmed from a 3-year-old *Callithrix jacchus*, i.e., a young adult, sexually mature healthy animal, raised in the self-sustaining marmoset monkey colony of the German Primate Center (Deutsches Primatenzentrum, DPZ, Göttingen, Germany). Samples were obtained as previously reported [30], following all legal regulations.

### 2.3. Treatment of HTPCs and MKTPCs with Dex

When confluence was reached, cells were starved for 24 h in serum-free medium to synchronize the cell cycle. Dexamethasone (Dex #1756; Sigma-Aldrich, Hamburg, Germany) was dissolved in ethanol. Cells were stimulated with Dex in a single dose at a concentration of 1 μmol/L as described before [8] for 24 h or 48 h; ethanol diluted in the medium at a final concentration of 0.04% (*v/v*) served as solvent control. This concentration of Dex is based on our previous study [8] and our pilot studies (not shown). It was also chosen because it is within the range of effective concentrations in patients [31].

### 2.4. Isolation of RNA, Reverse Transcription (RT-PCR) and Quantitative Real-Time PCR (Qpcr)

The isolation of RNA from HTPCs and complementary DNA (cDNA) synthesis were performed as recently described [8]. For qPCR measurements, cDNA was diluted adequately, and runs were performed with the QuantiFast SYBR Green PCR Kit (Qiagen, Hilden, Germany) on the LightCycler 96^®^ System (Roche Diagnostics, Penzberg, Germany) as recently outlined [8]. Primers (Table S1) were designed using the primer3 tool (http://primer3.wi.mit.edu.) (accessed on 11 January 2021), and samples were run in duplicate and analyzed using the ^ΔΔ^Cq calculation method [32]. Amplicons were ascertained by agarose gel electrophoresis and verified by sequence analysis (Eurofins, Ebersberg, Germany). For qPCR measurements, treatment with Dex (1 µmol/L) was performed for 24 h.

### 2.5. Western Blotting

For Western blot experiments, HTPCs were treated in a single dose with Dex (1 µmol/L) for 24 h or 48 h, as mentioned above. Protein sampling and determination, as well as separation and immunoblotting, were performed with whole-cell lysates as described recently [8]. Membranes were incubated overnight with the rabbit polyclonal anti-GR antibody (1:2500) as formerly used [8] or with the rabbit polyclonal anti-platelet derived growth factor receptor alpha antibody (PDGFRα; 1:1000; #3164; Cell Signaling Technology, Danvers, MA, USA) at 4 °C followed by IRDye800-labeled secondary antibodies for 1 h as described earlier [8]. Membranes were scanned with the infrared-based Odyssey Imaging System (Li-Cor, Bad Homburg, Germany) and visualized using the Image Studio software. To control for equal loading across all wells and for effective protein transfer during Western blotting, an anti-ACTB mouse monoclonal antibody (1:5000; #A5441, Sigma-Aldrich St. Louis, MO, USA) was applied, as well.

### 2.6. Cytokine Profiler Study

Supernatants of Dex-treated (1 µmol/L) versus untreated (DMEM + 0.0% (*v/v*) EtOH) cells were collected after 48 h, centrifuged for 3 min 8000 rpm and analyzed using the Proteome Profiler Human XL Cytokine Array Kit (#ARY005B; R&D Systems, Minneapolis, MN, USA) according to the manufacturer’s instructions. Membranes were scanned with the infrared-based Odyssey Imaging System, and analysis of spot density was performed using the Image Studio software (Version 5.2; LI-COR Biotechnology, Bad Homburg, Germany).

### 2.7. IL6 ELISA Measurements

Culture supernatants of Dex-treated (1 µmol/L) and untreated (DMEM + 0.04% (*v/v*) EtOH) control cells were harvested after 48 h and assessed for the presence of IL6 using the commercial Human IL6 Platinum Ready-to-Use Sandwich ELISA per manufacturer’s instructions (Affymetrix eBioscience, San Diego, CA, USA) as described earlier [16,33]. Cellular supernatant of control and Dex-treated cells were derived from 4 donors. Samples were run in duplicates, and values were normalized to cellular protein.

### 2.8. GR siRNA Studies

A total of 1.2 × 10^5^ cells were cultured in a 6-well plate overnight, as previously described [34]. Transfection was achieved using predesigned 10 nmol/L GR-siRNA (GR, #sc-35505, Santa Cruz Biotechnology, Dallas, TX, USA) or non-template (NT) control siRNA (10 nmol/L) with Lipofectamine RNA iMAX reagent (#13778150; ThermoFisher Scientific, Waltham, MA, USA) diluted in OPTI-MEM (#31985047, Gibco, ThermoFisher Scientific, Waltham, MA, USA), as described in the manufacturer’s instructions. In detail, the cell medium was replaced by DMEM with 1% (*v/v*) FCS, including ¼ of the transfection medium, and, 18 h after transfection, the medium was replaced by DMEM with 1% (*v/v*) FCS. At 48 h post-transfection, cells were either stimulated with 1 µmol/L Dex or 0.04% (*v/v*) ethanol, which served as the solvent (control) diluted in DMEM with 1% (*v/v*) FCS for 24 h. The efficacy and specificity of siRNA knockdown were verified at the protein level 48 h and 72 h after transfection using Western blot analysis or at the RNA level after 72 h, using real-time qPCR. After transfection, we studied the effect of GR siRNA knockdown on the gene expression of GR and two Dex-responsive genes, FKBP5 and IL6, via qPCR.

### 2.9. Statistical Analysis

Statistical analyses of qPCR and ELISA data were obtained using GraphPad Prism 6.0 Software (GraphPad Software Inc., San Diego, CA, USA). qPCR data were analyzed via a one-sample *t*-test of ^−ΔΔ^Cq values, as were cytokine levels in the supernatant via one-sample *t*-tests of the not-normalized values. The mRNA data derived from siGR RNA experiments are depicted as mean + SEM. A paired *t*-test was used to compare two groups. A probability value of *p* < 0.05 was considered significant and of *p* < 0.01 highly significant.

### 2.10. Sample Preparation for Proteome and Secretome Analysis

For the analysis of proteomes and secretomes, the HTPCs of 6 donors were used. A total of 10^6^ cells per sample were lysed in a buffer consisting of 8 mol/L urea (Carl Roth, Karlsruhe, Germany) in 50 mmol/L ammonium bicarbonate (Riedel-de Haën, Seelze, Germany) and sonicated in a Bandelin Sonoplus HD3200 cup resonator (Bandelin, Berlin, Germany) for a total of 15 min (in an alternating sequence of 20 s–pulse and 40 s–rest). Lysates were centrifuged through QIAshredder devices (QIAGEN, Hilden, Germany). Protein concentration was determined using the Pierce 660 nm assay reagent (Thermo Scientific, Waltham, MA, USA). From each sample, 10 µg were taken and 10X diluted using the above-mentioned lysis buffer. Cysteine residues were reduced for 30 min at 56 °C using 1,4-dithiothreitol at a concentration of 5 mM. Alkylation was performed for 30 min at room temperature by the addition of iodoacetamide to give a concentration of 15 mM, in darkness. Excess iodoacetamide was quenched by 1,4-dithiothreitol addition (final concentration 10 mmol/L) and a 15 min incubation at room temperature. The digestion of proteins was performed in two steps: (i) lysyl-endopeptidase C (Lys C; Fujifilm Wako, Neuss, Germany) 1:100 enzyme/protein ratio, for 4 h at 37 °C; (ii) the dilution of the samples with 50 mmol/L ammonium bicarbonate to 1 mol/L urea and overnight digestion with modified porcine trypsin (Promega, Madison, WI, USA) in a 1:50 enzyme/protein -ratio at 37 °C. For the secretome analysis, individual samples were concentrated using 3 K–2 mL Amicon centrifugal filters (Merck Millipore, Tullagreen, Carrigtwohill, Ireland). After the first step of volume reduction to approximately 50 µL, samples were adjusted to 1 mol/L urea in 50 mM ammonium bicarbonate and recentrifuged to reduce the volume to approximately 50 µL again. Concentration determination was performed as described above for cellular proteomes. Digestion was performed as described above for cellular proteomes without the dilution step in (ii).

### 2.11. Nano LC-MS/MS Analysis

Peptide samples were dissolved using the LC-eluent A (0.1% (*v/v*) formic acid in water). The analysis was carried out on an Ultimate 3000 RSLC instrument connected to a Q Exactive HF-X mass spectrometer (Thermo Scientific, Waltham, MA, USA). For proteome analysis, 1.5 µg, and for secretome analysis, 750 ng were loaded onto a trap column (Acclaim Pepmap™ 100,100 µm × 2 cm, C18, 5 µm, 100 Å, Thermo Scientific). The LC-separation of peptides was performed on an EASY-spray column (Pepmap™ RSLC C18, 2 µm, 100 Å, 75 µm × 50 cm, Thermo Scientific, Waltham, MA, USA) with the flow rate set to 250 nL/min. For the proteome analysis of cells incubated with Dex (1 µmol/L) for 24 h, a two-step gradient from 3% B (0.1% (*v/v*) formic acid in acetonitrile) to 25% B in 160 min followed by a ramp to 40% B for 10 min. For the proteome analysis of cells incubated with Dex (1 µmol/L) for 72 h, a two-step gradient from 6% B to 20% B in 80 min followed by a ramp to 40% B for 9 min was used. For the analysis of respective secretome sample sets (for both 24 and 72 h incubation time), a two-step gradient from 3% B to 25% B in 30 min followed by a ramp to 40% B in 5 min was applied. Mass spectrometry was performed in a data-dependent acquisition mode with a maximum of 15 MS/MS spectral scans per cycle.

### 2.12. Data Analysis and Bioinformatic Processing

Acquired MS spectra from each of the 4 separately processed datasets were processed using MaxQuant (1.6.11.0), with the “match between runs feature” and the label-free quantification feature being activated. Database search was performed using the *H. sapiens* subset of the Swiss-Prot (retrieval: 10/2020) and complemented by MaxQuant′s built-in contaminant database. Data analysis, volcano plot analysis, principal component analysis (PCA) and heatmaps were carried out with Perseus v1.6.5.0 [35] and R Statistical Software (v4.0.4; R Core Team 2021). For multiple testing correction and significance cut-off curve generation, the following parameters were used: s0 = 0.1 and FDR < 0.05. For protein annotation and functional enrichment analysis, the Database for Annotation, Visualization and Integrated Discovery (DAVID) [36], as well as the PROTEOMAPS tool, was used [37]. DAVID analysis was performed using the following categories: GO_MF_all (molecular function), GO_BP_all (biological process), GO_CC_all (cellular component), KEGG and Reactome pathways. The classification stringency of the analysis was set to high. The mass spectrometry data were deposited to the ProteomeXchange Consortium (www.proteomexchange.org, accessed on 3 October 2022) via the Proteomics Identification Database (PRIDE) partner repository with the dataset identifiers PXD033504 for both the 24 h and 72 h secretome datasets, PXD033514 for the 24 h proteome dataset and PXD033534 for the 72 h proteome dataset [38].

## 3. Results

### 3.1. Results of Cytokine Profiler Assay and IL6 ELISA

**Figure 1:**
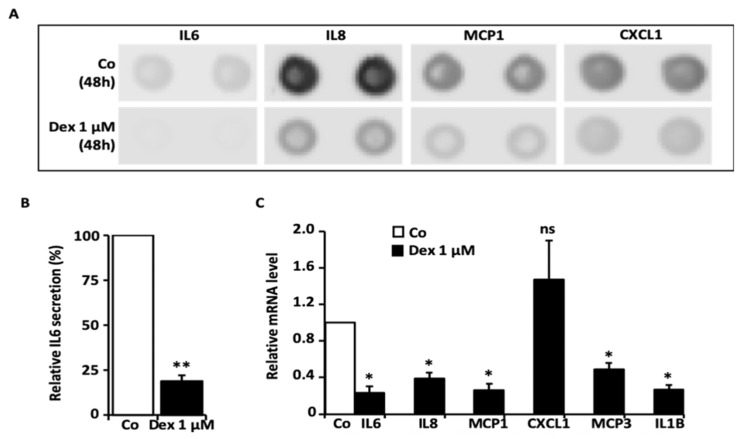
Consequences of Dex on cytokine levels. (**A**) HTPCs treated with 1 µM Dex for 48 h responded with the decreased secretion of IL6, IL8, MCP1 and CXCL1 compared to untreated cells (*n* = 1), as demonstrated by a human cytokine profiler assay. The upper panel shows the corresponding membrane spots of untreated control, while the lower panel depicts Dex (1 µM)-treated HTPCs and reveals a reduction of the signal intensity of IL6, IL8, MCP1 and CXCL1. (**B**) Using an ELISA, highly significantly decreased IL6 levels could be detected in the culture media of HTPCs from four individual patients treated for 48 h with 1 µM Dex compared to the corresponding control condition. Results were normalized to the total protein amount and statistically analyzed using a paired *t*-test (two-tailed paired *t*-test: ** *p* < 0.01; *n* = 4). (**C**) Quantitative PCR revealed significantly decreased mRNA expression levels of *IL6*, *IL8*, *MCP1*, *MCP3* and *IL1B* in HTPCs after 24 h stimulation with 1 µM Dex. *CXCL1* transcripts did not change significantly compared to control (ns: not significant, * *p* < 0.05; *n* = 4).

We screened the levels of cytokines in the culture media after a 48 h incubation time with 1 µM Dex, using a cytokine profiler assay (using HTPCs from one donor). Compared with basal conditions (control), 1 µM Dex markedly decreased proinflammatory cytokines, including IL6 (interleukin-6), IL8 (interleukin-8) and MCP1 (monocyte chemoattractant protein-1), along with CXCL1 (C-X-C motif chemokine ligand 1) (Figure 1A and Figure S1A). In order to test some of the changes, an ELISA for IL6 was implemented, and the results showed that the IL6 secretion from HTPCs stemming from four different donors was markedly and statistically significantly decreased after 48 h of 1 µM Dex (Figure 1B). Levels of *IL6* and additional factors were further examined by qPCR (using cells from four donors). Results showed that transcript levels of *IL6*, *IL8*, *MCP1* and *MCP3* (monocyte chemoattractant protein-3), as well as *IL1B* (interleukin-1beta), were significantly lower (Figure 1), while *CXCL1* did not change significantly upon 1 µM Dex.

Studies were also performed in the testicular peritubular cells of a nonhuman primate species, *Callithrix jacchus* (MKTPCs, *n* = 1). When exposed to 1 µM Dex for 24 h, the results showed that the mRNA of *IL6*, *IL8*, *IL1B* and *MCP1* were markedly decreased (Figure S1B). Taken together, these data indicate that Dex has an immunosuppressive role in both HTPCs and MKTPCs.

### 3.2. Consequences of the Downregulation of GR by Sirna

**Figure 2:**
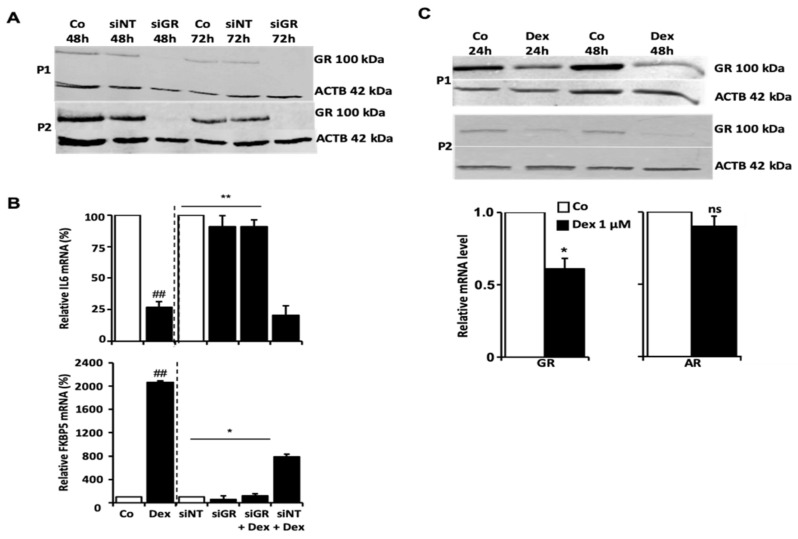
Regulation of GR by siRNA and Dex. (**A**) Silencing of GR by siRNA: Two representative GR Western blots from two patients (P1 and P2, *n* = 2) show the results of HTPCs transfected with non-targeting (NT) control siRNA and GR siRNA (siGR) at two different time points (48 h and 72 h) each and emphasize the efficiency of the transfection with siGR RNA. (**B**) Transfection of HTPCs with a GR siRNA abolished the observed effect on *IL6* and *FKBP5* mRNA. Quantitative PCR data of HTPCs from three different patients (*n* = 3) transfected with a non-targeting control siRNA (siNT) or GR siRNA (siGR) and treated with Dex (1 µM; siNT + Dex and siGR + Dex) or an equal volume of EtOH (Basal Co and Dex) for 24 h are depicted, ## *p* ≤ 0.01 vs. Co cells; * *p* ≤ 0.05, ** *p* ≤ 0.01 vs. siNT + dex cells. (**C**) Stimulation of HTPCs with Dex led to the downregulation of GR expression. In the presence of 1 µM Dex for 24 h and 48 h, GR protein in HTPCs from two different donors (P1 and P2, *n* = 2) was downregulated compared to control (Co), as shown by Western blot. In addition, significantly diminished *GR* (*n* = 7; upper left picture) but not *AR* mRNA (*n* = 9; lower left picture) was detected after 24 h incubation with 1 µM Dex.

We performed a transient knockdown of GR using siRNA in HTPCs. Non-targeting siRNA served as a control. HTPCs (from two different donors; P1; P2) were treated with 1 µM Dex for 24 h afterwards. As depicted in Figure 2A, the interference efficiency of GR expression was monitored using Western blotting. GR protein levels were reduced after 48 h and remained below the detection level for 72 h after siGR treatment. In the controls (no siGR RNA) or non-targeting siRNA (siRNA NT), the GR levels were not affected. Upon GR knockdown, the typical Dex-responsive gene *FKBP5* (an immunophilin and cochaperone belonging to the family of FK506-binding proteins [39]) and *IL6* were examined by qPCR. As shown in Figure 2B, *IL6* mRNA was strongly decreased (*p* < 0.01) by Dex, while a massive increase of *FKBP5* mRNA (*p* < 0.01) was measured in response to 24 h Dex exposure compared to control. These changes were abolished when siGR RNA was applied to cells 48 h before Dex treatment. Parallel studies with siRNA NT confirmed specificity.

### 3.3. Dex Decreased Levels of GR and Increased Levels of DKK1, ANG and DUSP1

We also examined whether Dex may affect the levels of the GR. We found that GR expression (transcript level at 24 h and protein level at 48 h) was substantially reduced by 1 µM Dex, whereas the mRNA levels of *AR* (androgen receptor) were not affected (Figure 2C). The evaluation of the Protein Profiler assay indicated that two proteins spotted on the cytokine array, namely Dickkopf-1 (DKK1) and Angiogenin (ANG), increased upon Dex. This result was in line with elevated mRNA levels, as shown by qPCR (Figure S2). Further, a more than 2.5-fold increase in Dual Specificity Protein Phosphatase 1 (*DUSP1)* mRNA was also detected after 24 h (Figure S2).

### 3.4. Unbiased Proteome and Secretome Analysis Reveal the Effects of Dex Treatment on Htpcs

For this approach, samples from *n* = 6 individuals were analyzed in *n* = 3 technical replicates per donor and experimental condition (i.e., for both Dex and Co), resulting in a total of 36 samples (18x Dex and 18x Co) per treatment duration (i.e., 24 h and 72 h). In total, 72 samples were analyzed by Nano-LC-MS/MS. For cells treated with Dex for 24 h, 3943 proteins and 48,239 peptides were identified, whereas 3017 proteins and 26,249 peptides were identified in cells treated for 72 h. A total of 467 proteins and 5765 peptides were identified in the secretomes of cells treated for 24 h and 380 proteins and 4153 peptides in the secretomes of cells treated for 72 h. All identified proteins, including the corresponding intensities, are listed in Table S2.

A principal component analysis (PCA) and unsupervised hierarchical clustering (Figure S3) revealed a separation between samples of individual donors, indicating differences between their proteome and secretome profiles. Interestingly, the PCA plot showed a separation according to treatment (Co vs Dex) within the six patients (Figure S3). Compared to the samples of 24 h Dex treated cells, the separation is more prominent after 72 h of treatment.

### 3.5. Differentially Abundant Proteins in the Proteome and Secretome of HTPCS after Dex Treatment

**Figure 3:**
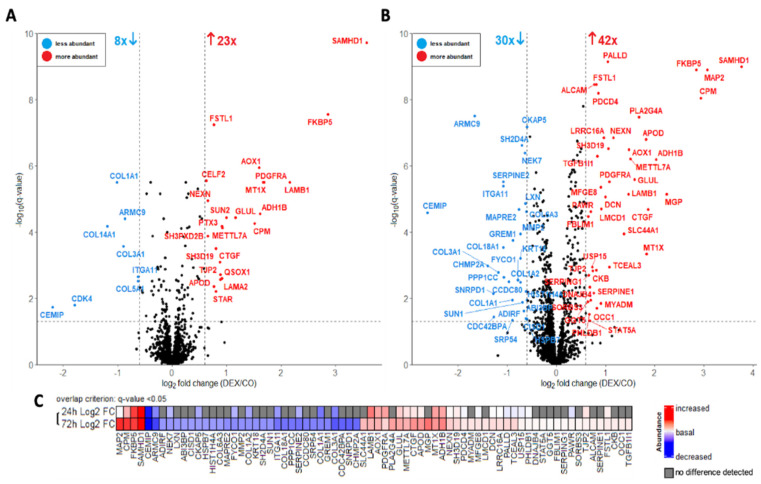
Volcano plot analysis of cellular proteomes treated with 1 µM dexamethasone for 24 h (**A**) and 72 h (**B**). The data was analyzed with a paired Student’s *t*-test; false-discovery rate (FDR): 0.05. Each colored dot represents a protein fulfilling the significance criteria (q-value < 0.05, log_2_ fold change >|0.6|): blue–proteins less abundant in the treated group; red–proteins more abundant in the treated group. For selected significant hits, gene names are displayed. (**C**) = log_2_ fold changes of significantly differentially abundant proteins of the 24 h-treatment and 72 h-treatment datasets. For the overlap, the 72 proteins (fulfilling the above significance criteria) from 72 h dataset were used as “base”. This was overlayed by the proteins from the 24 h dataset fulfilling only the “overlap criterion” of q-value < 0.05, which additionally includes potential trends, given the lower size of this dataset.

**Figure 4:**
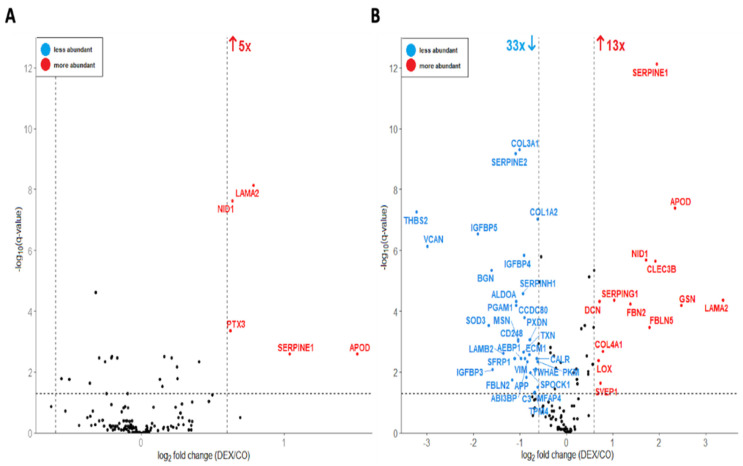
Volcano plot of cellular secretomes treated with 1 µM dexamethasone for 24 h (**A**) and a 72 h (**B**). The data was analyzed with a paired Student’s *t*-test; false-discovery rate (FDR): 0.05. Each colored dot represents a protein fulfilling the significance criteria (q-value < 0.05, log_2_ fold change > |0.6|): blue—proteins less abundant in the treated group; red—proteins more abundant in the treated group. For selected significant hits, gene names are displayed.

To identify differentially abundant proteins, a volcano plot analysis was performed using a paired Student’s *t*-test for the proteome (Figure 3) and secretome (Figure 4) datasets (24 h and 72 h Dex incubation) separately. Proteins were filtered and considered significant based on the following criteria: q-value (FDR-corrected *p*-value) < 0.05 and log_2_ fold change >|0.6|. This resulted in 31 proteins that were significantly altered in abundance in the 24 h dataset (8 less abundant and 23 more abundant in “Dex”, Figure 3A) and 72 significant proteins in the 72 h dataset (30 less abundant and 42 more abundant in “Dex”, Figure 3B). Furthermore, the differentially abundant proteins in the 72 h proteome and the 24 h proteome datasets showed considerable overlap, which is illustrated in Figure 3C.

The proteins that decreased in abundance after Dex treatment comprised multiple components of the extracellular matrix (ECM), such as collagens and collagen-interactors. Furthermore, several proteins related to the microtubule cytoskeleton were decreased.

**Figure A1:**
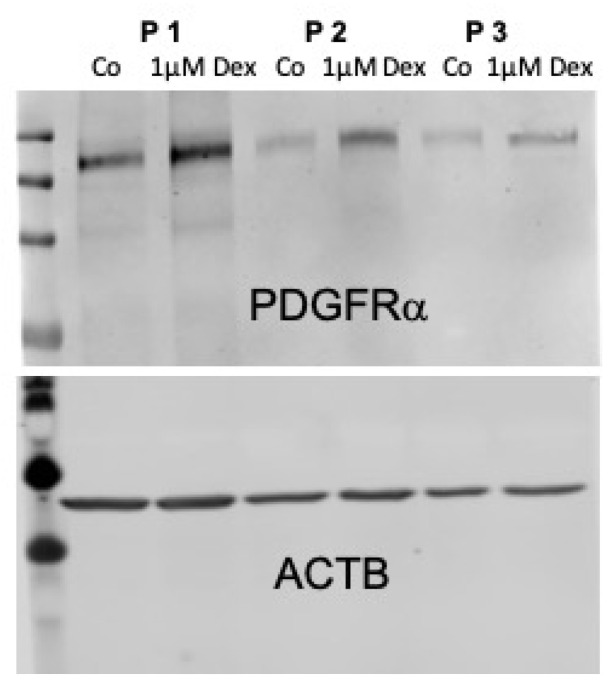
Regulation of PDGFRalpha (PDGFRa) by Dex. In the presence of 1 µM Dex for 72 h, PDGFRa protein in HTPCs from three donors (P1-P3) was increased, compared to controls (Co), as shown by Western blot.

The proteins with increased abundance included several components and interactors of the actin cytoskeleton. Furthermore, two proteins relevant for steroid hormone synthesis were increased in abundance, the steroidogenic acute regulatory protein (StAR) and the peptidyl-prolyl cis-trans isomerase FKBP5. Additionally, a series of growth factor proteins showed increased abundance, among them the transforming growth factor beta-1-induced transcript 1 protein (TGFB1I1) and connective tissue growth factor (CTGF), also known as CCN2 and platelet-derived growth factor receptor alpha (PDGFRα). The increased abundance of PDGFRα-protein upon the Dex treatment of HTPCs was confirmed by Western blot, providing an example for the robustness of the experimental data (also refer to: Appendix A Figure A1).

### 3.6. Bioinformatic Analysis of Significantly Changed Proteins Using DAVID and PROTEOMAPS

**Figure 5:**
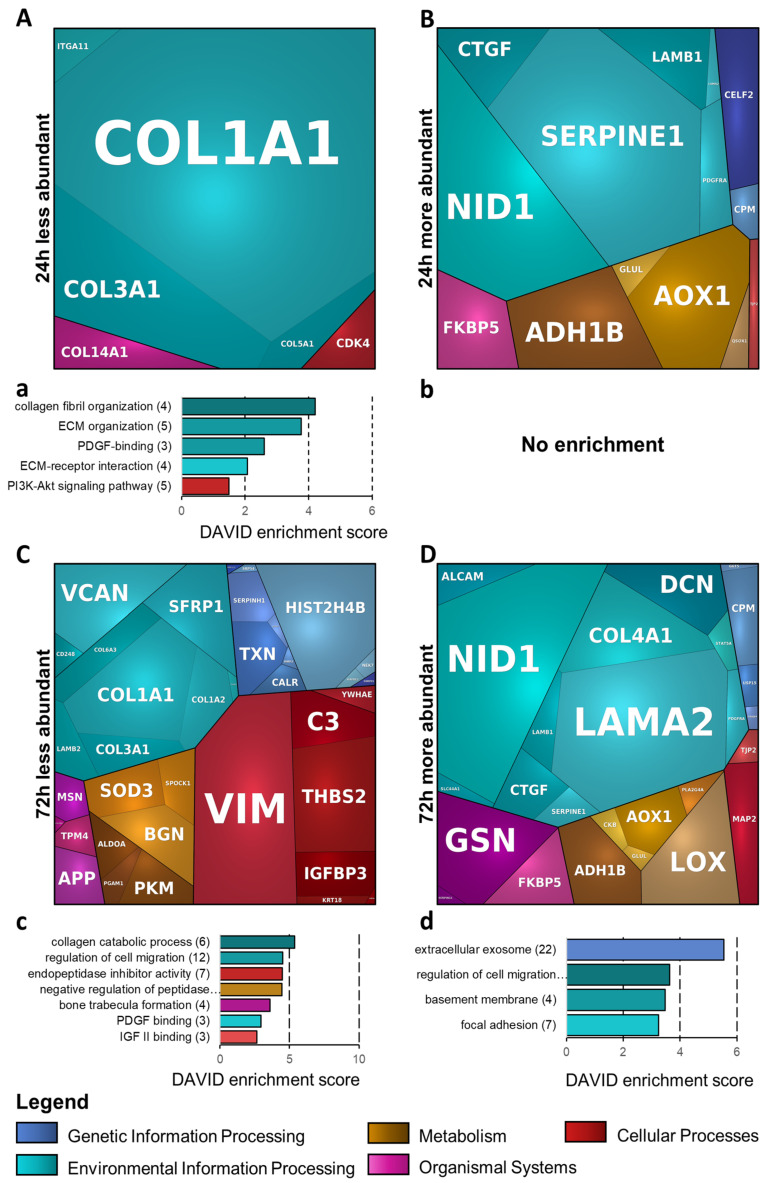
Bioinformatic analysis based on the functional enrichment of all merged (proteome + secretome) significantly differentially abundant proteins (**A**,**B**). 24 h-treatment, PROTEOMAPS-based analysis. (**a**,**b**): 24 h-treatment, DAVID-based analysis. (**C**,**D**): 72 h-treatment, PROTEOMAPS-based analysis. (**c**,**d**): 72 h-treatment, DAVID-based analysis. For PROTEOMAPS, proteins were annotated using GO “biological process”. The terms are shown in the legend and comprise all tiles of similar color. Annotated individual proteins are displayed in the mosaic plots. For DAVID analysis, functional enrichment terms GO_BP_all, GO_CC_all, GO_MF_all, KEGG and Reactome were used.

For bioinformatics analysis, the proteome datasets were merged with the respective secretome datasets. The differentially abundant proteins were analyzed using DAVID and PROTEOMAPS (Figure 5 and Table 1). The DAVID analysis of proteins decreased in abundance after 24 h Dex treatment (Figure 5a), displaying the GO-terms “collagen fibril organization” and “ECM organization”, containing collagen proteins, as most enriched. Furthermore, several of the proteins were co-annotated to the “PI3K-Akt signaling pathway”, among them collagens, cyclin dependent kinase 4 (CDK4) and integrin alpha-11 (ITGA11). In the PROTEOMAPS analysis, proteins altered in abundance after 24 h Dex were assigned to the terms “Environmental Information processing” (containing ECM proteins of the collagen and laminin groups, as well as a few of their interactors), “Organismal Systems” (including both steroid-hormone- and ECM-biosynthesis-related entries), “Metabolism”, “Cellular Processes” (with relevance to tight-junction formation), and “Genetic Information Processing” (Figure 5A,B).

In the subset of proteins decreased in abundance after 72 h, DAVID reflected an overrepresentation of proteins related to the terms “collagen catabolic process”, “endopeptidase inhibitor activity”, “PDGF binding” and “IGF II binding” (Figure 5c). In the subset of proteins that increased in abundance after 72 h, an overrepresentation of proteins related to the following terms was found: “extracellular exosome” (a rather general term, including most of the input entries), “regulation of cell migration” (including ECM- and actin-cytoskeleton-related proteins, as well as some growth factors), “focal adhesion” (comprising several actin-cytoskeleton-modulating and -interacting proteins) and “basement membrane” (containing laminin and collagen group proteins and their interactors) (Figure 5d).The corresponding PROTEOMAPS analyses displayed the terms “Environmental Information processing” (including the entire functional diversity of proteins identified here, similar to the above DAVID-term “extracellular exosome”), “Organismal Systems”, “Metabolism”, “Cellular Processes” and “Genetic Information Processing” (Figure 5C,D).

## 4. Discussion

The GR is expressed in the human testis, yet the possible roles of GCs in the regulation of the testis are not well examined. Within the human testis, peritubular cells are a predominant site of GR expression [7,8] and isolated HTPCs retain the expression of the GR, opening a window of opportunity to examine GC actions. This study identifies in a comprehensive way molecular changes induced by Dex in HTPCs.

Dex, acting at the GR, as well as further synthetic GCs, are widely used in medicine for the treatment of rheumatic and skin diseases, severe allergies and asthma, lung diseases and also Covid-19 infections [26,40,41,42]. The concentration of Dex used in our present and in a previous study [8], i.e., 1 µM, is within the therapeutic range [31]. Hence our results may bear potential clinical relevance.

### 4.1. Immunological Functions

Dex is known to suppress immune cell functions, in general [22], yet it is becoming clear that the response to GCs is highly cell-type-dependent. Responses differ both in terms of the individual genes and pathways affected, as well as in the magnitude of the transcriptional regulation and protein methylation of the GR [24,43,44,45,46,47]. HTPCs have immunological roles and secrete cytokines [16,17,18]. Hence, we performed a screening approach and explored whether Dex may affect cytokine secretion. Based on the results, we also performed ELISA and qPCR studies.

The results indicate, amongst others, that Dex reduces both the mRNA and protein levels of IL6. We performed the siRNA-mediated down-regulation of GR. The results confirmed that this action of Dex is mediated by GR. IL6, as a prototype inflammatory factor, has been shown to be involved in further, testis-specific functions. More specifically, it impairs the integrity of the blood–testis barrier and therefore could impair fertility [48]. A recent study showed that, in mice lacking IL6, this deficiency is associated with increases in sperm production and the testicular testosterone and dihydrotestosterone levels [49]. Interestingly, at the molecular level, five putative GR binding sites (GR1–5) were determined on the IL6 promotor, GR2 and GR3 of which are functional and essential binding sites for GR to modulate IL6 promotor activity [50]. Whether this mode of action also occurs in HTPCs requires additional studies, which go beyond the scope of this study.

The initial cytokine profiler approach and subsequent qPCR measurements indicated that other immunological responses are dampened by Dex, e.g., Specific roles in the human testis, going beyond immunological actions, are not well established, and therefore we did not follow up on these factors.

One consequence of Dex, namely that it significantly reduced GR levels in HTPCs, was also noted. It raises the possibility that the anti-inflammatory and other responses, initiated by Dex, may be weakened, especially by prolonged exposure to Dex. It is well known that GCs are able to downregulate GR levels in many but not all cell types, presuming a possible mechanism of GC resistance by lowering GR protein levels after prolonged exposure to GCs [44,51,52]. Clearly, whether the cellular data obtained in HTPCs can be transferred to the in situ situation in the testis of men remains to be tested. Nonetheless, the need for balanced and homeostatic GC levels for male fertility is highlighted by the reduced fertility reported in adrenalectomized rats [53] and by the first reported case of Addison‘s disease presenting as male infertility [6].

The cytokine profiler approach also revealed that, 48 h after Dex treatment, other proteins were altered, including DKK1 and ANG. Very little is known about the expression and functions of DKK1 and ANG in the testis and in the context of reproduction in general. ANG is a potent proangiogenic factor [54] e.g., in the ovary and in the human testis, specifically in peritubular myoid cells [55,56]. Dkk1 belongs to a family of four members (Dkk1–4) encoding secreted proteins that antagonizes the Wnt/beta-catenin regulated processes involved e.g., in embryonic development, adult tissue homeostasis and in tumors [57,58,59]. This secreted protein is involved in DEX-mediated hair follicle regression [60] and controls vascular smooth muscle cell proliferation and migration [61]. Whether it is also involved in the regulation of peritubular, smooth muscle-like cells remains to be studied.

### 4.2. Proteome and Secretome Changes Induced by Dex Treatment

Using a bottom-up mass spectrometry approach, 4410 and 3397 proteins (24 h and 72 h Dex treatment, respectively) were identified. Among them were numerous SMC-related markers and ECM proteins of the laminin and collagen families, as well as several candidates mediating immunological and inflammatory roles, which is in line with our recent findings and underlines the analytical depth of our analysis. Additionally, similar to our previous studies, among others, an enrichment of the following proteins in HTPC secretomes could be reproduced: FKBP5, FBLN2, FBLN5, FBN2 and LAMA2 [10], further demonstrating the validity of the presented datasets. The PCA analysis of the individual HTPC proteome and secretome profiles confirmed the previously described donor-specific heterogeneity [13,16,62]. Apart from donor-specific effects upon Dex treatment (e.g., increased levels of aortic smooth muscle actin 2 (ACTA2) in three patients), substantial common alterations in all patients were detected. These changes occurred in a time-dependent manner and were in general more pronounced after 72 h of exposure to Dex.

### 4.3. Dex Treatment Alters the Abundance of Cytoskeletal Proteins

Numerous proteins related to the actin cytoskeleton formation, organization and stability were increased after Dex, with stronger alterations after 72 h treatment. Among these proteins were Nexilin (NEXN), an F-actin binding protein, essential for SMC-like function [63,64], but Calponin 1 (CNN1) and Transgelin (TAGLN) also showed a similar tendency. As the NEXN promoter contains a GC-responsive sequence motif and as hydrocortisone treatment increases NEXN expression [65], a direct impact of Dex on NEXN may be the cause for the observed increase of ACTA2 in three of the involved patients. This is in line with earlier results demonstrating increased *ACTA2* expression from the Dex-mediated activation of HTPC-GR [8].

Interestingly, the F-actin uncapping protein (LRRC16A; also known as CARMIL1), a protein directly involved in promoting actin polymerization [66], and Palladin (PALLD), a microfilament-associated protein mainly expressed by SMCs [67] facilitating the assembly of highe- order actin structures, such as stress fibers or the formation of focal adhesions, were likewise increased. The mechanical tension generated within stress fibers (and with increased numbers of these structures) is known to promote the assembly of focal adhesions [68]. Interestingly, additional proteins related to focal adhesions (Figure 5d), such as GSN (Gelsolin), SORBS3 (Vinexin), FBLIM1 (filamin-binding LIM protein 1) and ALCAM (CD166 antigen), were also increased after Dex exposure [67,69,70,71,72]. While there is no HTPC-specific report to date, similar studies in the trabecular meshwork cells of the eye also demonstrated an increase in the crosslinking of actin networks as a key contributor to higher cellular elasticity and increased ECM stiffness [73,74,75], indispensable for tuning and modulating mechanical properties.

The increase of Fibrillin-2 (FBN2) and Fibulin-5 (FBLN5), essential for the assembly of microfibrils [76], as well as elastic fiber formation and organization thereby providing mechanical elasticity to tissues [77], is in line with our previous study showing that 24 h Dex stimulation of HTPCs leads to an increase in the mRNA levels of FBLN5 and Fibrillin-1 (FBN1) [8]. Of note, FBN1 was also more abundant in the present study (Log_2_ fold changes of 0.30 (q-value = 6.8 × 10^−6^) and 0.21 (q-value = 7.7 × 10^−5^), after 24 h and 72 h Dex treatment, respectively). In this regard, the elasticity of the HTPCs, which forms the wall of seminiferous tubules, is of utter importance for the wave-like contractility in the process of sperm transport [14,78]. In the past, testicular tubular atrophy has been attributed to disorders related to the formation of elastic fibers [79].

In contrast to the actin-related proteins discussed above, cytoskeletal proteins relevant for microtubule (MT) dynamics (polymerization and organization) were, in general, decreased upon Dex. For instance, the MT-associated proteins RP/EB family member 2 (MAPRE2) and KRT18 (keratin, type I cytoskeletal 18) become prominently visible among others (e.g., the cytoskeleton-associated protein 5 (CKAP5) or the LisH domain-containing protein ARMC9 (ARMC9)). Recent work has identified MAPRE2 as an important cell migration marker involved in the modulation of focal adhesion dynamics [80,81], which was significantly downregulated by Dex in a previous study [82]. KRT18 is a well-known component of the complementary cytoskeletal network [83] and, along with MAPRE2, can be influenced by gonadotropins [84]. While the role of the microtubule networks in the testis were addressed earlier [85], the specific functions of these proteins in HTPCs remain to be examined.

### 4.4. Dex Treatment Alters the Abundance of ECM-Related Proteins

HTPCs are an important source of ECM proteins in the testicular peritubular compartment. The ECM in this compartment shows pronounced signs of alterations during development and in cases of male infertility [8,16,29,86]. Our study indicates that Dex is a regulator of the abundance of collagens COL1A1, COL1A2, COL3A1, COL5A1 and COL14A1, as well as integrin alpha-11 (ITGα11), which is a collagen receptor protein [87], and collagen-associated interactors, as well as proteins mediating the degradation of collagen. Our finding is supplemented further by proteins related to “collagen fibril organization”, which is in line with the GR-dependent inhibition of collagen biosynthesis in GC-treated human fibroblasts and cells of the skin, lung and liver [88,89]. Another study reported that Dex exerts a negative effect on the steady-state levels of procollagen transcripts in mice [90].

Among further proteins found differentially abundant were some laminins (LAMA2, LAMB1, LAMB2) and the laminin-interacting protein Nidogen-1 (NID1), as well as proteins involved in the hyaluronan (HA) household maintenance of the ECM, CEMIP (cell migration-inducing hyaluronidase 1, also known as KIAA1199), a protein involved in hyaluronan catabolism [91], and PDGFR alpha (platelet-derived growth factor receptor alpha). Several studies indicated the increased HA degradation or increased levels of “trimmed” short-length HA fragments attributed to inflammatory/neoplastic diseases by promoting angiogenesis and cell migration [92,93]. However, the expression and function of CEMIP has not been reported for human testicular cells. One may speculate that the Dex-dependent decline of the CEMIP protein in HTPCs may be linked to an increased deposition of full-length HA to the ECM, which in turn could contribute to a decrease in HA fragment-mediated inflammatory processes. In this regard, our study also measured elevated levels of PDGFRα in HTPCs (also refer to: Appendix A Figure A1). According to Hammer et al., PDGFRα activation is linked to an increased deposition of HA into the mammary ECM [94].

Upon Dex treatment, we further found increased protein levels of pentraxin-related protein PTX3 (PTX3), which is in line with a previous work in human airway smooth muscle cells [95] but also of decorin (DCN). As previously shown [10], both proteins are expressed by HTPCs, are able to interfere with paracrine signaling and are linked to testicular inflammation and impaired spermatogenesis but also female fertility [16,86,95]. In addition, several other studies suggest them as essential–hormonal sensitive–multifaceted structural components of the ECM-modulating HA organization [96,97,98,99,100,101]. Collectively, it can be speculated that a drop of CEMIP abundance, concomitantly with an increase in PDGFRα, PTX3 and DCN release upon Dex stimulation, could have implications on HTPCs and the ECM matrix composition.

## 5. Conclusions

Our results identify novel actions of Dex in HTPCs. They include anti-inflammatory responses but also a downregulation of the GR, implying that such anti-inflammatory actions can be weakened upon exposure to this synthetic GC. The obtained proteome and secretome data further pinpoint massive changes in the cellular phenotype of HTPCs. If transferable to the human testis, such changes may also result in men upon treatment with Dex and may affect the cells and the ECM of the peritubular compartment, and thereby the overall testicular functions.

## Figures and Tables

**Table 1:** Summary of proteins altered in abundance upon dexamethasone treatment.

*Less Abundant Proteins (72 h merged dataset)*		
Enriched Term	DAVID Enrichment Score	Annotated proteins
IGF II binding	2.67	IGFBP5, IGFBP4, IGFBP3
PDGF binding	2.96	COL1A1, COL3A1, COL1A2
bone trabecula formation	3.59	GREM1, COL1A1, SFRP1, MMP2
negative regulation of peptidase activity	4.45	YWHAE, C3, APP, ECM1, LXN, SERPINE2, SERPINH1, SPOCK1, COL6A3
endopeptidase inhibitor activity	4.48	C3, APP, LXN, SERPINE2, SERPINH1, SPOCK1, COL6A3
regulation of cell migration	4.53	GREM1, COL1A1, COL18A1, COL3A1, ECM1, SFRP1, CEMIP, IGFBP5, SERPINE2, IGFBP3, MSN, CALR
collagen catabolic process	5.37	COL1A1, COL18A1, COL3A1, COL1A2, MMP2, COL6A3
*PROTEOMAPS—less abundant proteins (72 h merged dataset)*		
Enriched term	Annotated proteins	
environmental information processing	VCAN, CD248, COL6A3, SFRP1, COL1A1, COL1A2, COL3A1, LAMB2, ITGA11	
genetic information processing	PPP1CC, SERPINH1, TXN, CALR, MMP2, HIST2H4B, NEK7, MAPRE2, SNRPD1, HSPB7	
metabolism	SOD3, SPOCK1, BGN, ALDOA, PGAM1, PKM	
organismal system	MSN, TPM4, APP, COL18A1	
cellular processes	VIM, C3, THBS2, YWHAE, IGFBP3, KRT18, CHMP2A	
*More Abundant Proteins (72 h merged dataset)*		
Enriched term	DAVID enrichment score	Annotated proteins
focal adhesion	3.25	ALCAM, GSN, PALLD, FBLIM1, TGFB1I1, NEXN, SORBS3
basement membrane	3.48	LAMA2, COL4A1, LAMB1, NID1
regulation of cell migration	3.64	STAT5A, PDGFRA, GSN, LAMA2, SERPINE1, MYADM, NEXN, LAMB1, APOD, DCN
extracellular exosome	5.55	CPM, GSN, LAMA2, SLC44A1, SERPINE1, LAMB1, NID1, FSTL1, FBLN5, CLEC3B, ALCAM, DNAJB4, MGP, MYADM, SERPING1, AOX1, APOD, CKB, METTL7A, MFGE8, GLUL, FKBP5
*PROTEOMAPS—more abundant proteins (72 h merged dataset)*		
Enriched term	Annotated proteins	
environmental information processing	ALCAM, NID1, SLC44A1, CTGF, SERPINE1, LAMB1, LAMA2, COL4A1, DCN, STAT5A, PDGFRA	
genetic information processing	GGT5, CPM, USP15, DNAJB4	
metabolism	ADH1B, CKB, GLUL, AOX1, PLA2G4A, LOX	
organismal system	GSN, SERPING1, FKBP5	
cellular processes	TJP2, MAP2	

## Data Availability

The mass spectrometry data were deposited in the ProteomeXchange Consortium (www.proteomexchange.org, accessed on 3 October 2022) via the Proteomics Identification Database (PRIDE) partner repository with the dataset identifiers PXD033504–for both the 24 h and 72 h secretome datasets, PXD033514–for the 24 h proteome dataset and PXD033534–for the 72 h proteome dataset.

## Supplementary Materials

The following supporting information can be downloaded at: https://www.mdpi.com/article/10.3390/cells11193164/s1, Figure S1: Original data showing cytokine profiler membranes of HTPCs from one donor and mRNA results obtained by MKTPCs; Figure S2: Activation of GR by Dex affected DKK1 and ANG; Figure S3: Principal component analysis (PCA) of 6 patients’ cellular proteomes upon a 24 h (A) and a 72 h (B) incubation with 1 µM Dex, as well as respective secretomes upon a 24 h (C) and a 72 h (D) incubation with 1 µM Dex (per patient: 3x Dex vs. 3x Co); Table S1. Primer sequences (5´–3´) used in real time PCR, amplicon, annealing temperature (AT) and accession number, *Callithrix jacchus* (*cj*); Table S2, lists S1–S8. Every two lists: all identified proteins and differentially abundant proteins, respectively, in the proteomes/secretomes of DEX-treated and control samples of all the experimental subsets.
