# Supplementary material for: Profound Effects of Dexamethasone on the Immunological State, Synthesis and Secretion Capacity of Human Testicular Peritubular Cells
Source: Cells. 2022 Oct 9;11(19):3164. doi: 10.3390/cells11193164 (PMC9562650; doi:10.3390/cells11193164)
Supplement: Supplementary file 1 [file cells-11-03164-s001.zip › cells-1842134 - supplementary.pdf]

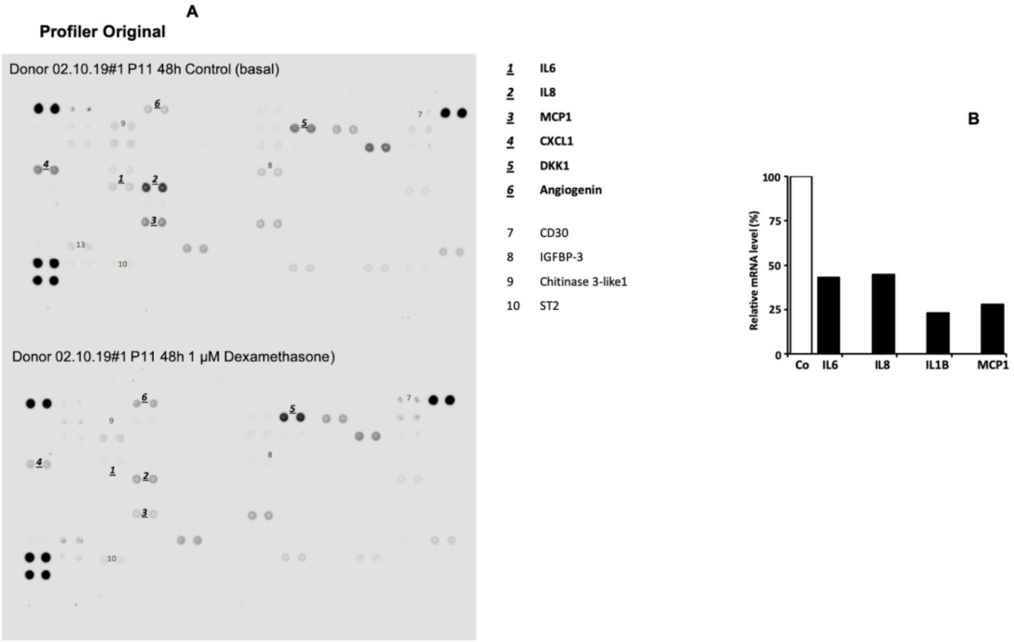

Figure S1. Original data showing cytokine profiler membranes and results in MKTPCs. (**A**) HTPCs treated with 1 µM Dex for 48 h responded with a decreased secretion of IL6, IL8, MCP1 and CXCL1 compared to untreated cells. Upper panel shows corresponding membrane spots of untreated control while lower panel depicts Dex (1 µM) treated HTPCs revealing a reduction of signal intensity of IL6, IL8, MCP1, CXCL1, chitinase 3-like 1 and IGFBP3 whilst dot intensity was increased for ANG, DKK1, ST2 and CD30. Dex = dexamethasone, Co = control (EtOH). Note that the dots in the lower left corners are reference spots used for normalization. (**B**) Quantitative PCR revealed decreased mRNA expression of *IL6*, *IL8*, *IL1B* and *MCP1* also in MKTPCs after 24 h stimulation with 1 µM Dex compared to control (n = 1).

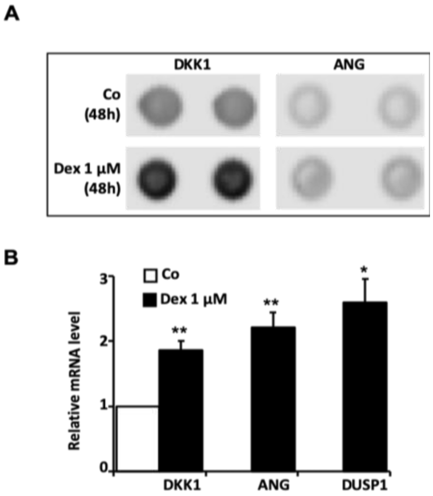

Figure S2. Activation of GR by Dex affected DKK1 and ANG. (**A**) Cultured HTPCs treated with 1 µM Dex for 48 h exhibited an increased secretion of DKK1 and ANG compared to untreated cells as revealed by a human cytokine profiler assay (n = 1). (**B**) Quantitative PCR corroborated significantly increased mRNA expression of *DKK1* and *ANG* (n = 6; $p < 0.05$) but also of *DUSP1* (n = 4) upon 24 h stimulation with 1 µM Dex. * $p \leq 0.05$, ** $p \leq 0.01$ vs. control cells. Dex = dexamethasone, Co = control (EtOH).

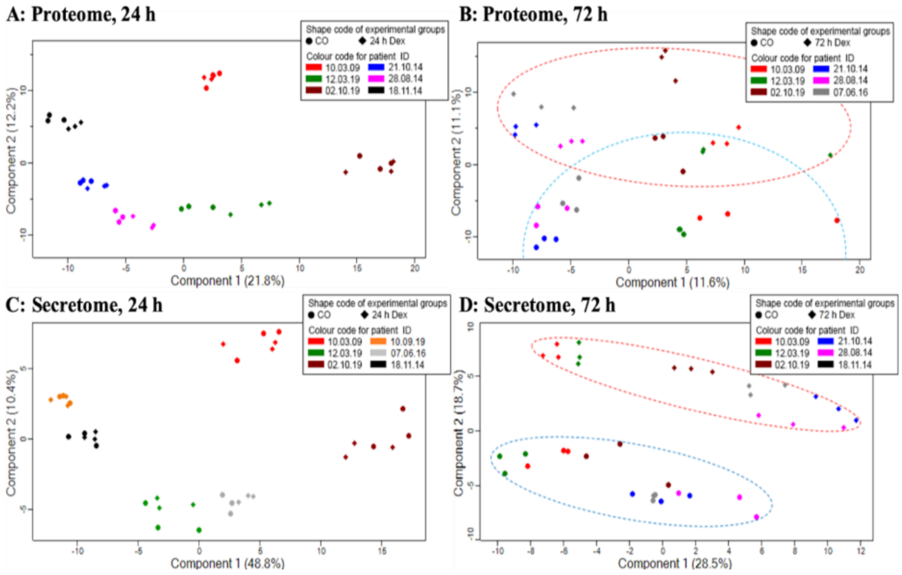

Figure S3. Principal component analysis (PCA) of 6 patients' cellular proteomes upon a 24 h (**A**) and a 72 h (**B**) incubation with 1 µM Dex, as well as respective secretomes upon a 24 h (**C**) and a 72 h (**D**) incubation with 1 µM Dex (per patient: 3x Dex vs 3x Co). Different dot colors encode the respective patient ID (a 6-digit code). Dex = dexamethasone, Co = control (EtOH).

Table S1. Primer sequences (5´-3´) used in real time PCR, amplicon, annealing temperature (AT) and accession number, *Callithrix jacchus* (cj).

Gene Name		Sequence (5´- 3´)	Amplicon (bp)	AT (°C)	Accession Number
RPL19	*For*<br>*Rev*	AGGCACATGGGCATAGGTAA<br>CCATGAGAATCCGCTTGTTT	199	59	NM_000981.3
FKBP5	*For*<br>*Rev*	GCATTATCCGGAGAACCAAA<br>GCCACATCTCTGCAGTCAAA	121	59	NM_004117.3
IL6	*For*<br>*Rev*	AACCTGAACCTTCCAAAGATGG<br>TCTGGCTTGTTCCTCACTACT	159	62	NM_000600.4
IL8	*For*<br>*Rev*	TCTTGGCAGCCTTCCTGA<br>GAATTCTCAGCCCTCTTC	271	60	NM_000584.4
IL1B	*For*<br>*Rev*	CTTGGTGATGTCTGGTCCATATG<br>GGCCACAGGTATTTTGTCATTAC	127	60	NM_000576.2
MCP1	*For*<br>*Rev*	AGGTGACTGGGGCATTGAT<br>GCCTCCAGCATGAAAGTCTC	109	58	NM_002982.3
MCP3	*For*<br>*Rev*	TGGAGAGCTACAGAAGGACCA<br>GTGGGGTCAGCACAGATCTC	94	58	NM_006273.3
CXCL1	*For*<br>*Rev*	CGCCCAAACCGAAGTCATAG<br>CTCTGCAGCTGTGTCTCTCT	211	60	NM_001511.3
DKK1	*For*<br>*Rev*	CCTTGGATGGGTATTCCAGA<br>CACAGTCTGATGACCGGAGA	96	60	NM_012242.4
ANG	*For*<br>*Rev*	GGCGTTTTGTTGTTGGTCTT<br>AGTGCTGGGTCAGGAAGTGT	97	60	NM_001145.4
DUSP1	*For*<br>*Rev*	CTGCCTTGATCAACGTCTCA<br>CTCCAGCATTCTTGATGGAG	153	60	NM_004417.4
GR	*For*<br>*Rev*	GAAGGAAACTCCAGCCAGAA<br>GATGATTTCAGCTAACATCT	159	60	NM_000176.3
AR	*For*<br>*Rev*	AGCCTCAATGAACTGGGAGA<br>TCCTGGAGTTGACATTGGTG	175	62	NM_000044.6
cjIL6	*For*<br>*Rev*	AAGAGGTAGCTGCCCCAAAT<br>AGTGCCTCTTTGCTGCTTTC	145	60	XM_017975106.1
cjIL8	*For*<br>*Rev*	ACAAGAGCCAGCAAGAAACG<br>CAGTGTGGTCCCCTCTCAAT	233	60	XM_002745733.3
cjIL1B	*For*<br>*Rev*	GGTTGTCGTGGCTATGGAGA<br>TTTTGTTGTGCATCCCGGAG	198	58	XM_008980284.3
cjMCP1	*For*<br>*Rev*	GCAGCAAGTGTCCCAAAGAA<br>TGGGGTTATGGAGTGAGTGT	154	60	XM_002748333.4
